# Effects of a high-phosphorus diet on the gut microbiota in CKD rats 

**DOI:** 10.1080/0886022X.2021.2003207

**Published:** 2021-12-03

**Authors:** Guoxin Ye, Wei Yang, Zhaori Bi, Liya Huang, Fang Liu

**Affiliations:** a Department of Geriatrics, Xinhua Hospital Affiliated to Shanghai Jiao Tong University School of Medicine, Shanghai, China; b School of Health Economics and Management, Nanjing University of Chinese Medicine, Nanjing, China; c National Clinical Research Center for Aging and Medicine, Huashan Hospital, Fudan University, Shanghai, China

**Keywords:** Chronic kidney disease, gut microbiota, high-phosphorus diet, serum phosphate, hypertension

## Abstract

**Objective:**

To investigate whether high-phosphorus diets alter gut microbiota in healthy rats and chronic kidney disease (CKD) rats.

**Methods:**

In this 4-week randomized controlled trial, healthy rats and CKD rats were fed a regular-phosphorus (Pi: 0.8%) and high-phosphorus (Pi: 1.2%) diet. The subjects were divided into four groups: sham-group rats with regular-phosphorus diet intervention (CTL group), sham-group rats with high-phosphorus diet intervention (CTLP group), CKD model rats with regular-phosphorus diet intervention (CKD group), and CKD model rats with high-phosphorus diet intervention (CKDP group). The V3-V4 region of the 16S rRNA gene was sequenced to study the effect of a high-phosphorus diet on gut microbiota.

**Results:**

A high-phosphorus intervention increased systolic blood pressure (SBP) and parathyroid hormone (PTH) in CTL and CKD rats but did not change serum creatinine and 25(OH)D levels. After the high-phosphorus diet, serum phosphate and fibroblast growth factor 23 (FGF23) increased in the CKDP group compared with the CKD group. The gut microbiota was significantly altered after intervention with a high-phosphorus diet in CTL and CKD group rats. A high-phosphorus diet reduced the Shannon index values of gut microbiota in all rats. The Chao1 and Ace indexes were decreased in the CTL group after high-phosphorus diet intervention. Some microbial genera were elevated significantly after high-phosphorus dietary intervention, such as *Blautia* and *Allobaculum*. The main bacteria linked to SBP and FGF23 also correlated directly with creatinine. After high-phosphorus diet intervention, the bacteria *Prevotella* were positively related to SBP in CTLP and CKDP groups.

**Conclusions:**

High-phosphorus diets were associated with adverse changes in gut microbiota and elevated SBP, which may have adverse consequences for long-term health outcomes.

## Introduction

Phosphorus is an essential element required for a variety of physiological functions in the human body. A 70-kg adult absorbs approximately 1.6 *g* of phosphorus daily from food. Approximately 40–60% of the total ingested phosphorus in food is absorbed by the small intestine, and the rest is excreted from feces. Phosphorus excretion occurs mainly through urine, regulated by a network consisting of phosphatonins, such as parathyroid hormone (PTH), fibroblast growth factor 23 (FGF23), Klotho, vitamin D3, thereby maintaining phosphorus homeostasis [1]. However, dietary phosphorus intake seemingly continues to increase as a result of the increasing application of phosphorus additives to processed foods and soft drinks [2]. As reported previously, 10–30% of the phosphorus consumed by people may be derived from inorganic phosphorus additives [2,3], exceeding the needs of healthy individuals. Although it is controversial whether dietary phosphorus intake results in direct serum phosphate changes in a healthy population, the potential adverse consequences to cardiovascular and bone health have received attention [4]. In individuals with chronic kidney disease (CKD), hyperphosphatemia is a common complication that leads to severe morbidity and poor outcomes [3]. In addition, CKD patients may be particularly vulnerable to high dietary phosphorus intake, and high phosphorus intake may accelerate CKD [5,6]. Similar results have also been found in animal experiments [7,8].

In humans, the gut microbiota has the largest numbers of bacteria and the greatest number of species [9]. The composition of human gut microbiota changes over time, when the diet changes, and as overall health changes [9]. Researchers found that the four dominant bacterial phyla in the human gut are *Firmicutes*, *Bacteroidetes*, *Actinobacteria*, and *Proteobacteria* [10]. Most bacteria belong to the genera *Bacteroides*, *Clostridium*, *Faecalibacterium*, *Eubacterium*, *Ruminococcus*, *Peptococcus*, *Peptostreptococcus*, and *Bifidobacterium*, other genera, such as Escherichia and *Lactobacillus*, are present to a lesser extent [11].

Recent studies have demonstrated that the interactive relationship between the host and gut microbiota in CKD populations and uremic toxins from the gut microbiota also contribute to CKD development [12]. There are significant changes in the gut microbiota of uremic patients compared with those of healthy controls [13,14]. Phosphorus is also a common uremic toxin. The treatment with phosphate binders could change intestinal microenvironments and alter gut microbiota communities [15]. However, it is surprising that little data are available on phosphorus intake by gut microbiota, especially in individuals with CKD.

This study aimed to investigate the effects of a high-phosphorus diet on gut microbiota in sham-group and CKD group rats; the results may provide valuable data for dietary nutrient management in both healthy and CKD populations and further provide potential treatment targets for CKD patients fed high-phosphorus diets.

## Material and methods

### Animals and dietary intervention

Male Sprague-Dawley rats (Slack Laboratory Animal Co., Ltd, China), weighing approximately 180–200 g, were housed in a temperature-controlled environment (20 °C) with a 12-h light/dark cycle in the experimental animal room at Shanghai Medical College of Fudan University. Rats were fed a regular-phosphorus diet (Pi: 0.8%) and drank freely. After a one-week adaptation period, the rats were randomly subjected to 5/6 nephrectomy or sham operation as described by Anderson [16]. Twelve weeks after surgery, blood samples were collected for creatinine measurements. Rats were divided into four groups: sham groups receiving the regular-phosphorus diet (Pi: 0.8%, *n* = 5, CTL) or the high-phosphorus diet (Pi: 1.2%, *n* = 7, CTLP) and CKD group receiving the regular-phosphorus diet (Pi: 0.8%, *n* = 7, CKD) or the high-phosphorus diet (Pi: 1.2%, *n* = 6, CKDP). The dietary interventions lasted for four weeks. The diet compositions of the two kinds of feed are summarized in Supplemental Table S1. The protocol was approved by the ethics committee for animal research of Fudan University (China).

Blood samples were collected at the beginning and end of the dietary intervention. Fresh feces were also collected. Both plasma and fecal samples were stored in a −80 °C freezer. Before and after the intervention, systolic blood pressure was measured in all rats (BP-2000; Visitech Systems).

### Blood analyses

The biochemical variables were measured by an automatic biochemical analyzer (HITACHI 7600-020), including creatinine, serum phosphate, and serum calcium. Serum levels of PTH, FGF23, Klotho, and 25(OH)D were assessed by ELISA kits (Immutopics, San Clemente, CA; IBL, MN, USA; Immunodiagnostic System, Boldon, UK).

### DNA extraction and 16S rRNA gene sequencing

DNA isolation and next-generation sequencing were performed on stool samples for each diet group (CTL, CTLP, CKD, and CKDP). The total DNA was extracted by bead beating and using an E.Z.N. A.R Stool DNA Kit (Omega Bio-tek, Inc., GA). In summary, 0.2 *g* of feces were thawed and added 790 mL lysis buffer containing 4 M guanidine thiocyanate, 10% N-lauroyl sarcosine, 5% N-lauroyl sarcosine-0.1 M phosphate Buffer [pH 8.0], and 1 *g* glass beads (0.1 mm, Biospec Products, Inc., USA). Use a vortex to mix the sample thoroughly. Beading at full speed is then carried out for 10 min. Extract DNA according to the E.Z.N.A.R stool DNA kit and follow the manufacturer's instructions. The V3-V4 region of the bacterial 16S rRNA gene was amplified from fecal genomic DNA using 341 F (5′-CCTACGGGNGGCWGCAG-3′) and 805 R (5′-GACTACHVGGGTATCTAATCC-3′) primers. Polymerase chain reaction amplification (PCR) was performed for 21 cycles with the following steps: 94 °C for 3 min, 94 °C for 30 s, 58 °C for 40 s, 72 °C for 1 min, and a final extension step of 72 °C for 5 min. The PCR products were then electrophoresed on a 2% agarose gel to confirm amplification. Products from different samples were indexed, mixed in equal proportions, and sequenced using the Illumina MiSeq platform (2 × 300 bp) according to the manufacturer's instructions.

### Bioinformatic analysis of 16S rRNA gene amplicons

Sequencing read pairs were demultiplexed based on unique molecular barcodes, and reads were merged using USEARCH V8.0 (http://drive5.com/usearch/). Merging allows an overlap of 0 mismatches and at least 50 bases. Sequences that could not be spliced and chimeras were eliminated by using UCHIME software. It was removed that sequences < 400 bases after splicing. Operational taxonomic units (OTUs) were clustered by using UPARSE [17] Version 7.1 software based on 97% similarity. The phylogenetic affiliation of each 16S rRNA gene sequence was analyzed by the RDP CLASSIFIER (http://rdp.cme.msu.edu/) using a confidence threshold of 70%. Additional analyses were performed using the QIIME 1.9 pipeline [18].

### Statistical analysis

The continuous data were expressed with medians and interquartile ranges. The nonparametric Kruskal-Wallis test was used for multiple group comparisons. The nonparametric Mann-Whitney *U* test was used for significant differences between the two groups, and the chi-square test was used for the comparison of categorical data. All *P*-values were adjusted for multiple comparisons through using the FDR algorithm, and *P*-values less than 0.05 after multiple comparison adjustments were considered significant. The principal coordinate analysis (PCoA) of the microflora data ordination plot was based on Bray-Curtis distances, and the significance among the four groups was determined by ANOSIM using the vegan package to compare the global microbiota composition before and after intervention in the four groups at the operational taxonomic unit (OTU) level. Linear discriminant analysis (LDA) combined with the effect size (LEfSe) algorithm was used to identify characteristic biomarkers [19]. Microbiome statistics and graph generation were performed using the ggplot2 software package [20]. Spearman rank correlation tests were performed to analyze the association of metadata with different bacterial genera in serum and feces. All statistical analyses were performed by using R 4.0 [21].

### Data availability

Raw sequencing data have been submitted to the NCBI Sequence Read Archive with accession number PRJNA701137.

## Results

### Biochemical variables

Serum creatinine, systolic blood pressure (SBP), and phosphatonins (FGF23, PTH, Klotho, and 25(OH)D) were higher in the CKD group than in the CTL group (*p* < 0.05), but there was no significant difference in serum phosphate and serum calcium between CTL and CKD groups (Table 1). The high-phosphorus intervention increased PTH and SBP levels in both CTL and CKD rats (*p* < 0.05) but did not change serum creatinine and 25(OH)D levels. After the high-phosphorus diet, serum phosphate and FGF23 increased significantly in the CKDP group compared with the CKD group.

**Table 1.** Variables of CTL and CKD group rats after different kinds of phosphorus diet intervention.

	CTL (*N* = 5)	CTLP (*N* = 7)	CKD (*N* = 7)	CKDP (*N* = 6)	*P* value (CTL vs. CKD)	*P* value (CTL vs. CTLP)	*P* value (CKD vs. CKDP)
Weight (g)	611.00 [545.00–636.00]	523.00 [504.00–528.50]	464.00 [449.50–473.00]	511.50 [484.00–542.75]	0.005	0.106	0.073
Creatinine (umol/L)	61.00 [59.00–70.00]	61.00 [59.00–63.50]	145.00 [132.00–147.50]	138.50 [123.75–158.50]	0.006	0.684	0.943
SBP (mmHg)	118.50 [114.50–124.60]	131.70 [129.88–132.30]	147.00 [143.25–155.80]	169.55 [162.82–174.17]	0.003	0.005	0.022
Serum phosphate (mmol/L)	2.56 [1.91–2.64]	2.55 [2.42–2.74]	2.39 [2.12–2.80]	3.15 [3.00–3.40]	0.876	0.745	0.035
Serum calcium (mmol/L)	2.08 [1.88, 2.41]	2.64 [2.58, 2.75]	2.32 [2.14, 2.70]	2.68 [2.56, 2.81]	0.106	0.030	0.101
PTH (pg/mL)	71.12 [51.16, 101.79]	235.51 [202.81, 310.35]	147.91 [125.22, 183.61]	1026.46 [719.43, 1463.92]	0.010	0.003	0.001
FGF23 (pg/mL)	14.99 [11.08, 17.27]	13.68 [10.60, 17.43]	25.99 [21.82, 31.59]	40.39 [38.56, 55.53]	0.003	0.755	0.001
Klotho (pg/mL)	14.68 [14.23, 17.30]	12.37 [10.26, 13.73]	13.24 [11.67, 13.76]	11.96 [10.75, 13.30]	0.048	0.005	0.366
25(OH)D (nmol/L)	103.55 [82.80, 104.93]	95.47 [87.99, 99.52]	57.86 [50.76, 59.33]	59.36 [46.24, 78.34]	0.003	0.639	0.628

Note: Data are presented as medians and interquartile ranges in squared parentheses and were tested using the Mann-Whitney U test. The adjusted *P* values were calculated by the P.adjust function in R using the "BH" method to control for the false discovery rate. Abbreviations: SBP: systolic blood pressure; PTH: parathyroid hormone; FGF23: fibroblast growth factor 23. Conversion factors for units: Creatinine in µmol/l to mg/dl, 0.01131; serum phosphate and serum calcium in mmol/l to mg/dl, 3.1.

### Analysis of the fecal microbiota

**Figure 1.**
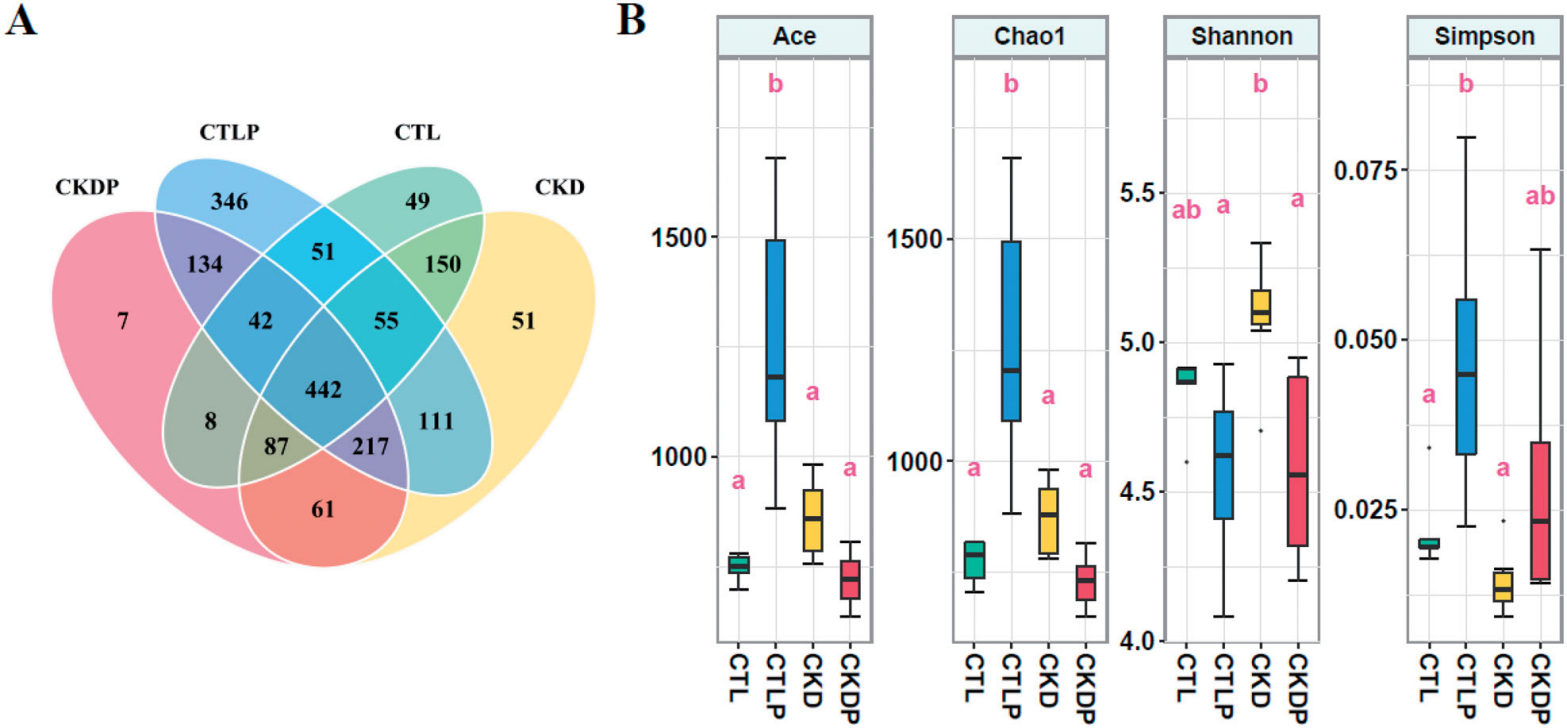
Gut microbial diversity of the CTL, CTLP, CKD, and CKDP groups. (A) Rarefaction analysis between the number of samples and OTUs. The overlaps displayed by the Venn diagram showed that 442 of the total number of 1846 OTUs were shared in the four groups. (B) Diversity estimation of the 16S ribosomal RNA gene library of the CTL, CTLP, CKD, and CKDP groups. Boxplots that do not share a letter are significantly different (*p* < 0.05).

**Figure 2.**
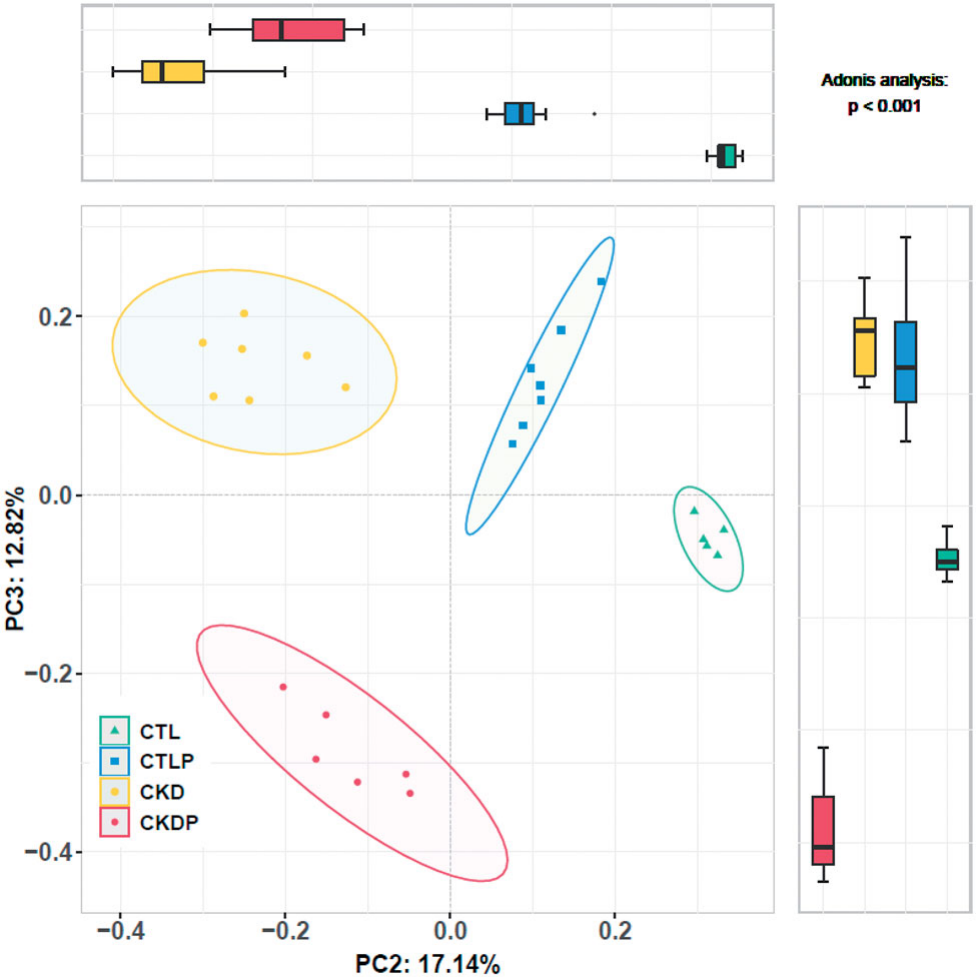
Overview of the unconstrained (unsupervised) ordination analysis of the CTL, CTLP, CKD, and CKDP groups. Principal component analysis plots based on Bray-Curtis distances showed that gut microbiota composition was significantly different among the four groups. The upper and right boxplots showed the difference between PC2 and PC3 among the four groups.

Rarefaction analysis showed that a high-phosphorus diet significantly increased OTUs in the CTLP group (Figure 1(A)). As shown in the Venn diagram, the total abundance of OTUs was 1864, and the four groups shared 442 OTUs. Notably, 346 OTUs were unique to CTLP, whereas only 7 OTUs were unique to CKDP. In addition, 49 OTUs were unique to the CTL group, and 51 OTUs were unique to the CKD group. The Chao1 estimator and the ACE estimator were used to calculating community richness. The Shannon calculator and the Simpson calculator returned community diversity indices for OTU definition. The Chao1 and Ace estimators were significantly increased in CTLP compared to CTL (Figure 1(B)), which meant the community richness was increased in the CTLP group. However, the Shannon index was significantly reduced after the high-phosphorus diet intervention, and the Simpson index was significantly increased after the intervention (Figure 1(B)). It indicated that community diversity was reduced after high-phosphorus diet intervention. We further performed a PCoA analysis to investigate potential differences, and we observed statistically significant differences among the four groups (Figure 2).

**Figure 3.**
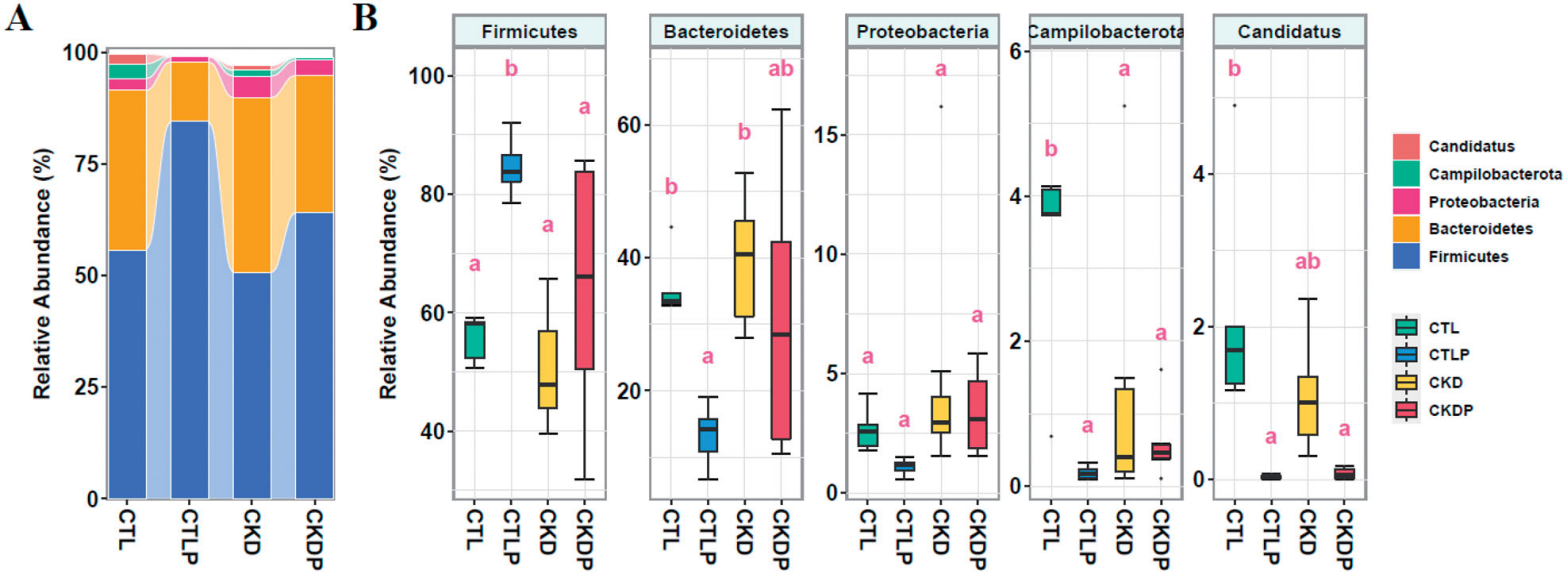
Taxonomic composition of the gut bacteria at the phylum level in the CTL, CTLP, CKD, and CKDP groups. (A) The gut microbiota of the four groups was dominated by *Firmicutes*, followed by *Bacteroidetes, Proteobacteria, Campilobacterota, and Candid*atus. (B) Box plots showed the abundance of different phyla in the four groups. Pairwise comparisons were performed using the 'multcomp' package in R, and a compact letter display was drawn for all pairwise comparisons. Boxplots that do not share a letter are significantly different (*p* < 0.05).

In all samples, the most represented phyla were *Firmicutes* and *Bacteroidetes*, followed by *Proteobacteria*, *Campilobacterota*, and *Candidatus* (Figure 3(A)). At the phylum level, increased *Firmicutes* and decreased levels of *Bacteroidetes* and *Candidatus* were observed in the CTLP and CKDP groups compared to the CTL and CKD groups (Figure 3(B)). In addition, *Campilobacterota* was significantly higher in the CTL group than in the other three groups.

### LEfSe analysis

**Figure 4.**
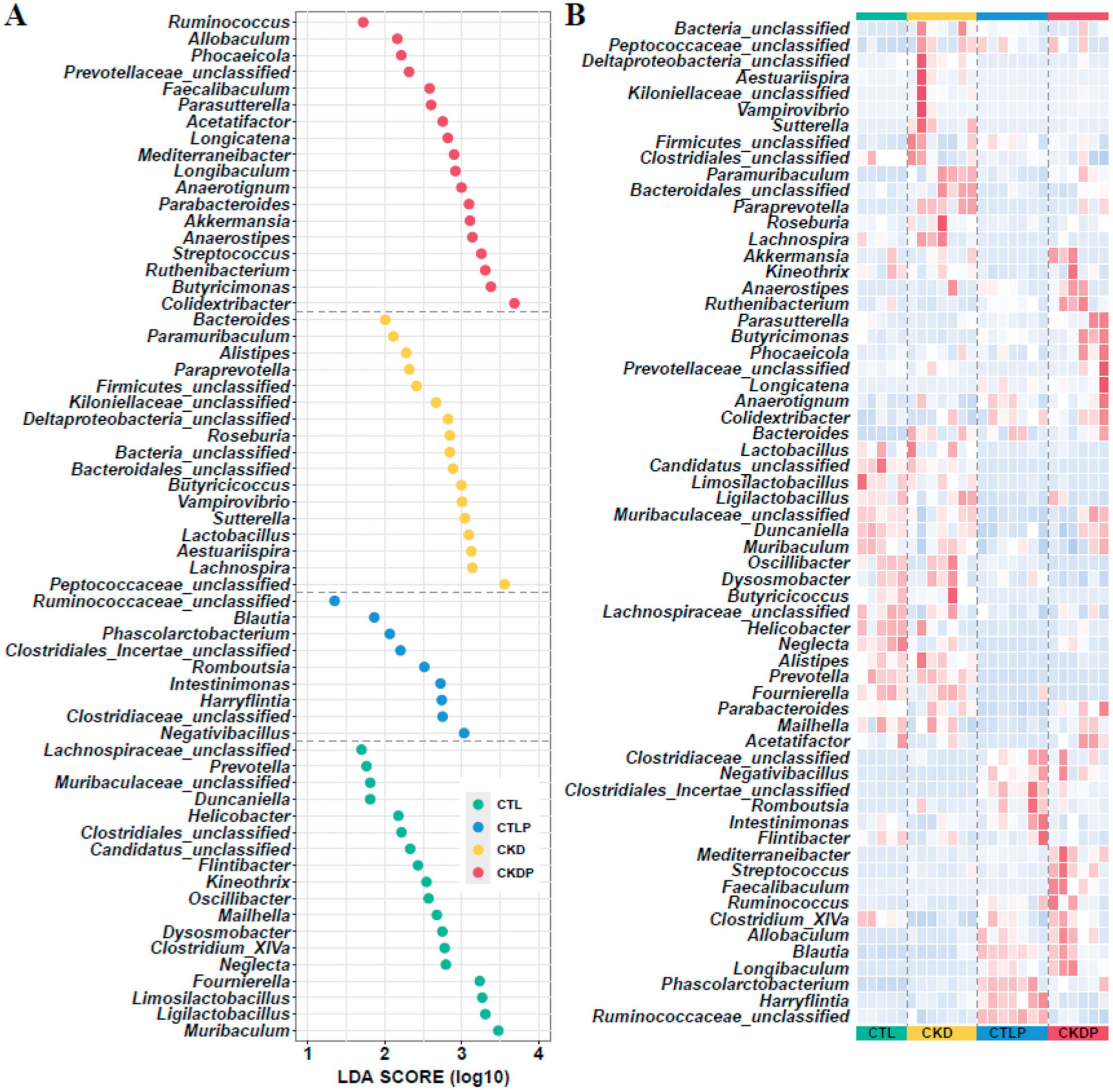
Discrepant analysis at the genus level of the CTL, CTLP, CKD, and CKDP groups. (A) LEfSe analysis identified the predominant taxa of each group. Green represents group-specific bacteria in the CTL group, yellow represents group-specific bacteria in the CKD group, blue represents group-specific bacteria in the CTLP group, and red represents group-specific bacteria in the CKDP group. (B) Heatmap and hierarchical clustering of the taxonomic genera are shown in Figure 5(A). LDA scores were calculated by LDA effect size using linear discriminant analysis to assess the effect size of each differentially abundant bacterial taxon.

The PCoA and the composition of phylum analyses showed significant differences among the four groups. LEfSe analysis was applied to detect the most differentially abundant taxa. A total of 61 genera were identified as group-specific sets. Based on LDA selection, the CTL group, the CTLP group, the CKD group, and the CKDP group were significantly enriched with 18 genera, nine genera, 17 genera, and 18 genera, respectively (Figure 4(A)). These prominently different microbial genera are also shown in the heatmap (Figure 4(B)). It should be noted that some genera significantly enriched in CTLP also had higher abundances in CKDP, such as *Blautia* and *Allobaculum*.

**Figure 5.**
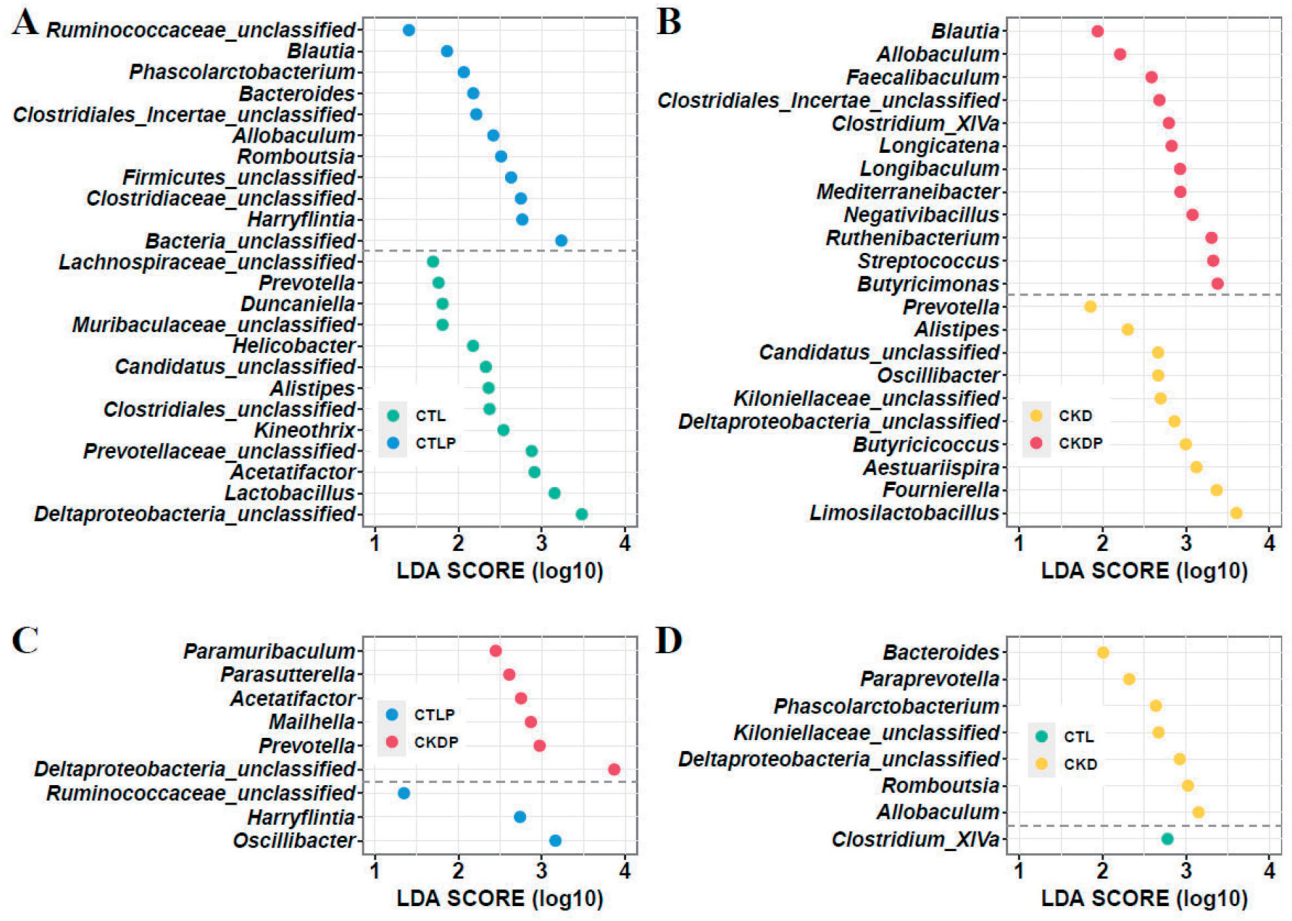
LDA effect size (LEfSe) analysis was performed to identify the taxa displaying the differences in abundance in the gut microbiota between groups. (A) Showed the CTL and CTLP groups, (B) showed the CKD and CKDP groups, (C) showed the CTLP and CKDP groups, and (D) showed the CTL and CKD groups. LDA scores were calculated by LDA effect size using linear discriminant analysis to assess the effect size of each differentially abundant bacterial taxon.

To further examine the impact of high-phosphorus dietary intervention on gut microbiota, we performed LEfSe analysis between the two groups. As shown in Figure 5, *Blautia*, *Allobaculum,* and *Clostridiales_Incertae_unclassified* were significantly increased after high-phosphorus intervention in both sham groups (Figure 5(A), CTL vs. CTLP) and CKD group rats (Figure 5(B), CKD vs. CKDP). In addition, *Harryflintia* and *Ruminococcaceae_unclassified* were significantly enriched in the CTLP group (Figure 5(A,C)). It should be noted that *Allobaculum*, which was significantly elevated by the high-phosphorus diet intervention, was also significantly elevated in the CKD group (Figure 5(D), CTL vs. CKD).

### Correlation analysis of gut microbiota, blood pressure and phosphatonins

**Figure 6.**
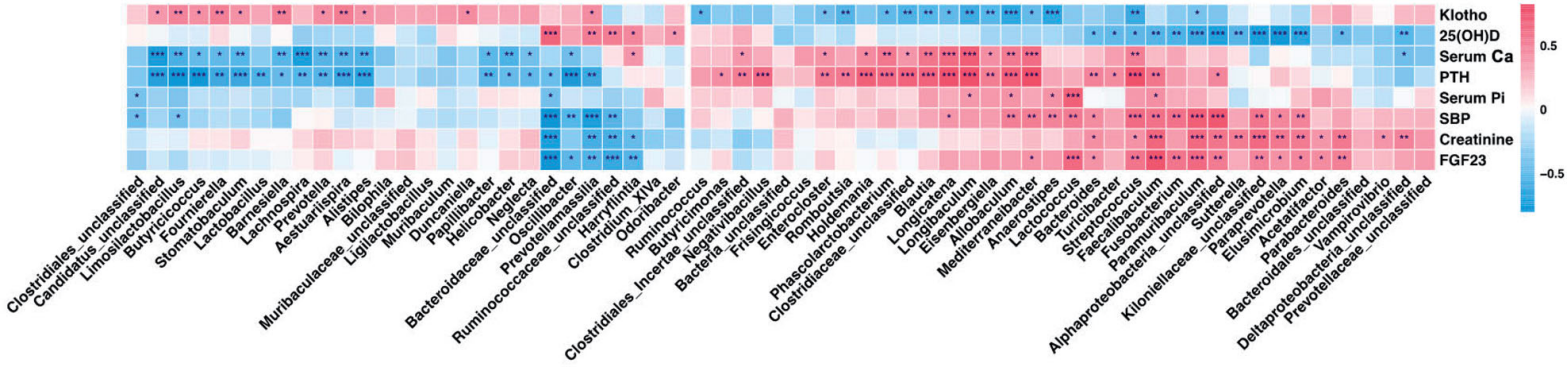
Correlation between SBP, phosphatonins (FGF23, PTH, Klotho and 25(OH)D), and gut microbiota. The number in each cell is the Spearman correlation coefficient, red indicates a positive correlation, and blue indicates a negative correlation.

We performed Spearman correlation analysis to characterize the correlation among gut microbiota, SBP, and phosphatonins (FGF23, PTH, 25(OH)D, and Klotho). The results indicated that the bacteria which enriched in the CKD group, CTLP group, and CKDP group (such as *Allobaculum*) were positively correlated with the levels of PTH, serum calcium, serum phosphate and SBP, negatively correlated with Klotho; bacteria that were enriched in the CTLP and CKDP group (such as *Blautia*) were positively correlated with PTH and serum calcium, and negatively related with Klotho. In addition, the main bacteria linked to creatinine and FGF23 also correlated directly with SBP, such as *Bacteroides*, *Faecalibaculum* (Figure 6).

### Correlation analysis of serum phosphate, blood pressure and gut microbiota in four groups

**Figure 7.**
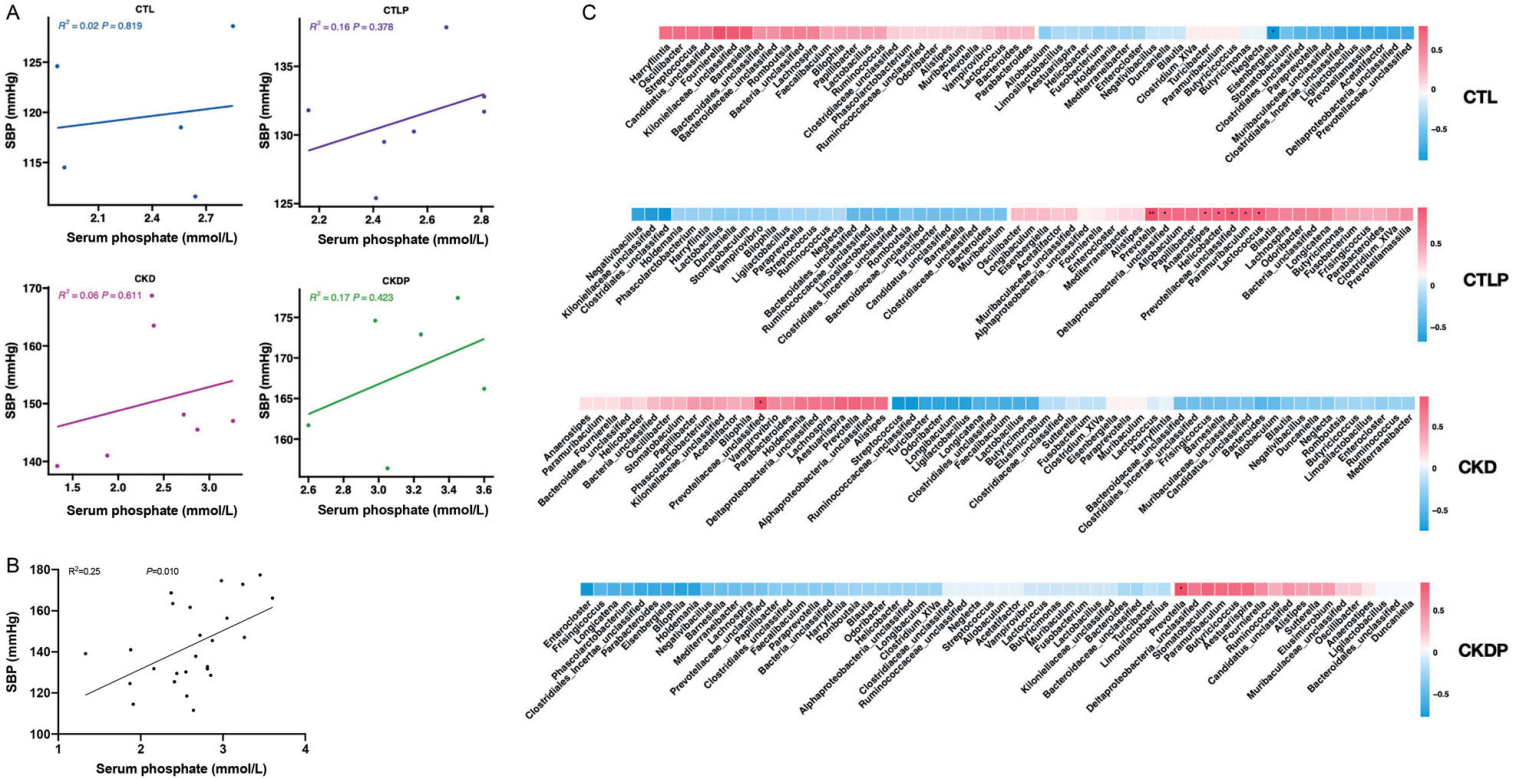
Correlation analysis of serum phosphate, blood pressure, and gut microbiota in four groups. (A) Pearson correlation analysis analyzed the relationship between SBP and serum phosphate in CTL, CTLP, CKD, and CKDP groups, respectively. (B) Pearson correlation analysis analyzed the relationship between SBP and serum phosphate in all groups (CTL + CTLP + CKD + CKDP groups). (C) The relationship between SBP and gut microbiota in four groups. Abbreviations: SBP, systolic blood pressure.

To further analysis the high-phosphorus diet intervention’s effect on blood pressure and gut microbiota, we analyzed the relationships between SBP, serum phosphate, and gut microbiota in four groups of rats. The SBP was not related to serum phosphate in each group (Figure 7(A)). Due to limited samples, we analysis the relationships between serum phosphate and SBP in all the rats. It suggested that SBP was positively associated with serum phosphate in all rats (Figure 7(B)). Figure 7(C) showed that some bacteria were positively related to SBP in CTLP and CKDP groups, such as *Prevotella.*

## Discussion

The high-phosphorus intervention increased SBP and PTH in CTL and CKD rats but did not change serum creatinine and 25(OH)D levels significantly. After the high-phosphorus diet, serum phosphate and FGF23 increased significantly in the CKDP group compared with the CKD group. The gut microbiota was significantly altered after high-phosphorus diet intervention. Notably, a high-phosphorus diet reduced the Shannon index values of the gut microbiota in all rats. The Chao1 and Ace indexes were significantly decreased in the sham group after high-phosphorus diet intervention. The high-phosphorus diet intervention groups had lower *Bacteroidetes* and *Candidatus* abundances (CTL vs. CTLP and CKD vs. CKDP). The effect of a high-phosphorus diet on the gut microbiota in the sham group and CKD group rats was typical and characteristic, and the elevated gut microbiota was similar, such as *Blautia* and *Allobaculum*. In addition, the main bacteria linked to creatinine and FGF23 also correlated directly with SBP. After high-phosphorus diet intervention, the bacteria *Prevotella* were positively related to SBP in CTLP and CKDP groups.

The diversity of gut microbiota is affected by many factors; however, it is no doubt that diet is one of the most important determiners of gut microbiota. Different nutritional strategies, such as a low-protein diet, have been proposed to regulate gut microbiota to reduce uremic toxins, which is beneficial for CKD patients [22]. However, few studies have been conducted on how dietary components, such as phosphorus. Phosphorus is a vital element in microbial and host metabolism, and it can also be a barrier against pathogens in the gut [23]. Whereas, a long-term high-phosphorus diet is harmful, whether in healthy or CKD populations [15,24–27]. Although the correlation between phosphorus metabolism and gut microbiota has been confirmed [13], research on the effect of a high-phosphorus diet on CKD gut microbiota is rare and should not be ignored due to the increasing application of phosphorus additives in daily food.

In our study, a high-phosphorus diet was used in daily feed in the sham group and CKD group rats directly. We observed that CKD rats had increased gut microbial diversity. The high-phosphorus diet increased the microbial community richness in the sham group but decreased the community diversity in both the sham group and CKD group rats. These results remind us that CKD alters gut microbiota and that variations in dietary phosphorus influence the disturbed gut microbiota in CTL and CKD rats. Moreover, the high-phosphorus diet significantly altered the gut microbiota in both CTL and CKD rats, with each group having specific microbial genera. The abundances of gut microbiota were similar in the CTL and CKD groups, as well as in the CTLP and CKDP groups, indicating the significant effects of a high-phosphorus diet on the gut microbiota distributions. This reminds us of the importance of phosphorus-restricted diet management for not only CKD patients but also healthy populations.

We also found that SBP increased in CTL and CKD rats after a 4-week high-phosphorus diet intervention. And high-phosphorus di*et al*so elevated FGF23 levels in the CKD group. It indicated that a high-phosphorus diet can induce the occurrence of hypertension and lead to an increase in FGF23. When dietary phosphorus loading, the increased serum levels of FGF23 promote phosphorus excretion from the kidney, which precedes a rise in PTH and serum phosphate, therefore maintaining the phosphorus homeostasis in the early stage of CKD. In healthy individuals with normal renal function, the increased SBP might be associated with a phosphate-specific increase in sympathoadrenergic activity [24]. In CKD, several studies showed that increased FGF23 or serum phosphate was closely associated with hypertension [28]. The prevalence of hypertension is elevated, and renal function declines in CKD patients [29]. The potential mechanisms are as follows: 1) FGF23 contributes to hypertension development by modulating the renin-angiotensin-aldosterone system [30]; 2) FGF23 regulates renal sodium reabsorption by up-regulating Na^+^ Cl^−^ cotransporters in renal tubules [31], and 3) high phosphorus increases renin expression and Ang II through PTH, thereby inducing hypertension [32]. Although many studies have been performed, the effects of dietary phosphorus intervention on gut microbiota and the correlation with blood pressure are still poorly understood. Next, we analysis the correlations between SBP, creatinine, and phosphatonins (FGF23, PTH, Klotho, and 25(OH)D) with the gut microbiota by using the Spearman correlation analysis. The results indicated that the gut microbiota correlations with SBP, creatinine, and FGF23 were almost the same. The bacteria, *Prevotell,* were positively related to SBP in CTLP and CKDP groups.

Recent research indicates that the gut microbiota has an essential role in hypertension development [33]. Gut microbiota dysbiosis has been reported in animal models and patients with hypertension. For example, in fecal microbiota transplantation experiments, fecal samples transferred from hypertension patients to germ-free mice increased blood pressure [34]. Moreover, studies have reported that interventions targeting the gut microbiota, such as probiotics, have effects on reducing the blood pressure [35,36]. The potential mechanisms are still unclear. The influence of gut microbiota on the host may be partially explained by the generation of short chain fatty acids (SCFAs), including the beneficial SCFAs (acetate, butyrate, and propionate) and the non-beneficial lactate [37]. These SCFA acting on cell surface receptors, including G protein-coupled receptor (GPR) 41, GPR43 and olfactory receptor 78 regulate blood pressure [37]. Gut microbiota can also influence the state of immunity and inflammation, cell metabolism, and proliferation that may eventually affect blood pressure [37]. As gut microbiota, dysbiosis is correlated with hypertension, and hypertension is an important factor that contributes to CKD development, the finding that the changes of the gut flora associated with impairment of renal function are not surprising. Although the high-phosphorus diet might directly affect the imbalance of gut microbiota, our finding suggested that the high-phosphorus diet resulted in gut dysbiosis which may be partially regulated *via* elevated SBP.

In this study, we did not observe microbial genera significantly associated with serum phosphate. The possible explanations are as follows. First, the digestion and absorption of inorganic phosphorus (sodium phosphate) added to feed is independent of the gut microbiota. Phosphorus absorption in the intestine depends on two mechanisms: an active process mediated by the Slc34a2 Na^+^/phosphate cotransporter NaPi-IIb [38] and a passive process that proceeds paracellularly [39]. Second, phosphorus homeostasis is a balance between intestinal absorption, renal excretion, and an internal contribution from the bone. Phosphatonins interact with each other to keep serum phosphate in the normal range [1]. Therefore, during a high-phosphorus diet, the body will activate various mechanisms to maintain serum phosphate stability. However, we did observe significant changes in gut microbiota induced by a high-phosphorus diet intervention. Therefore, the dynamic changes in bacteria during intervention with a high-phosphorus diet require further investigation.

Some limitations should be noted. First, we did not test inflammatory factors. The gut microbiota *Blautia,* elevated after the high-phosphorus diet intervention, was positively associated with inflammatory factors [40], which might be involved in the inflammatory state in healthy individuals or CKD. Thus, further investigation is required to determine whether changes in *Blautia* affect phosphorus intake by modulating inflammatory states in CKD populations. Second, the small sample size leads to large volatility of the data. Since it was a preliminary experiment, further investigations are needed to enlarge the sample size and explore other parameters, making it possible to evaluate the mechanism of how a high-phosphorus di*et al*tered the gut microbiota and may provide a new target for intervention of hyperphosphatemia.

In conclusion, the long-term high-phosphorus diet increased SBP and altered gut microbiota dysbiosis in healthy and CKD rats. The high-phosphorus diet may directly alter the intestinal microbiota profiles, or partially affect the gut microbiota *via* elevated SBP. The phosphorus-restricted diet management is of great importance for both CKD patients and healthy populations. In addition, further studies on the mechanism of phosphorus on gut microbiota are needed to provide precise dietary guidance for phosphorus management of CKD patients.

## Supplementary Material

Supplementary Material
