# Supplementary material for: Effects of a high-phosphorus diet on the gut microbiota in CKD rats
Source: Ren Fail. 2021 Dec 3;43(1):1577–87. doi: 10.1080/0886022X.2021.2003207 (PMC8648004; doi:10.1080/0886022X.2021.2003207)
Supplement: Supplementary Material [file IRNF_A_2003207_SM3565.pdf]

Supplementary material for Ye et al., “Effects of a High-phosphorus Diet on the Gut Microbiota of CKD rats”, *Renal Failure*, 2021

Supplemental Table S1. Diet composition.

	Regular-phosphorus diet	High- phosphorus diet
Composition	Content (g / Kg)	Content (g / Kg)
Tyrosine	200	200
L-cystine	3	3
Corn starch	388	388
Maltodextrin	151.20	151.20
Saccharose	100	100
Cellulose	45	45
Soya-bean oil	50	50
$CaHPO_4$	44.50	66.66
Fat	10	10
$CaCO_3$	2	0
Choline	2.50	2.50
Salt	2.50	2.50
Mineral Mix	1.30	1.30
Total (g)	1000	1017.66
Phosphate%	0.80	1.20
Calcium%	1.01	1.39
Fat%	5	5

Note: Diets were formulated and produced by Research Diets Inc (Shanghai Fanbo Biotechnology Co., Ltd.).
